# Image-Guided Radiotherapy Using a Modified Industrial Micro-CT for Preclinical Applications

**DOI:** 10.1371/journal.pone.0126246

**Published:** 2015-05-19

**Authors:** Manuela C. Felix, Jens Fleckenstein, Stefanie Kirschner, Linda Hartmann, Frederik Wenz, Marc A. Brockmann, Gerhard Glatting, Frank A. Giordano

**Affiliations:** 1 Medical Radiation Physics/Radiation Protection, Universitätsmedizin Mannheim, Medical Faculty Mannheim, Heidelberg University, Mannheim, Germany; 2 Department of Radiation Oncology, Universitätsmedizin Mannheim, Medical Faculty Mannheim, Heidelberg University, Mannheim, Germany; 3 Department of Neuroradiology, Universitätsmedizin Mannheim, Medical Faculty Mannheim, Heidelberg University, Mannheim, Germany; 4 Department of Diagnostic and Interventional Neuroradiology, University Hospital Aachen, Aachen, Germany; University of Nebraska Medical Center, UNITED STATES

## Abstract

**Purpose/Objective:**

Although radiotherapy is a key component of cancer treatment, its implementation into pre-clinical in vivo models with relatively small target volumes is frequently omitted either due to technical complexity or expected side effects hampering long-term observational studies. We here demonstrate how an affordable industrial micro-CT can be converted into a small animal IGRT device at very low costs. We also demonstrate the proof of principle for the case of partial brain irradiation of mice carrying orthotopic glioblastoma implants.

**Methods/Materials:**

A commercially available micro-CT originally designed for non-destructive material analysis was used. It consists of a CNC manipulator, a transmission X-ray tube (10–160 kV) and a flat-panel detector, which was used together with custom-made steel collimators (1–5 mm aperture size). For radiation field characterization, an ionization chamber, water-equivalent slab phantoms and radiochromic films were used. A treatment planning tool was implemented using a C++ application. For proof of principle, NOD/SCID/γc^−/−^ mice were orthotopically implanted with U87MG high-grade glioma cells and irradiated using the novel setup.

**Results:**

The overall symmetry of the radiation field at 150 kV was 1.04±0.02%. The flatness was 4.99±0.63% and the penumbra widths were between 0.14 mm and 0.51 mm. The full width at half maximum (FWHM) ranged from 1.97 to 9.99 mm depending on the collimator aperture size. The dose depth curve along the central axis followed a typical shape of keV photons. Dose rates measured were 10.7 mGy/s in 1 mm and 7.6 mGy/s in 5 mm depth (5 mm collimator aperture size). Treatment of mice with a single dose of 10 Gy was tolerated well and resulted in central tumor necrosis consistent with therapeutic efficacy.

**Conclusion:**

A conventional industrial micro-CT can be easily modified to allow effective small animal IGRT even of critical target volumes such as the brain.

## Introduction

External beam radiotherapy (RT) is a mainstay of cancer treatment. However, its implementation into pre-clinical (small) animal models involving small target volumes is frequently omitted due to a high technical complexity associated with multi-angle or multi-planar irradiation to spare healthy tissue [1–9]. For example, in malignant glioma, which resembles a highly aggressive form of brain tumors, partial-brain RT is an integral part of the current standard of care [10, 11]. The easiest approach to implement irradiation in a pre-clinical glioma model would be to use a clinical LINAC. Although modern machines can be easily employed to treat very small intracerebral lesions in humans, brain tumor xenografts in animals as small as mice will be below the detection limit of on-board positioning (cone-beam) CTs or even clinical CT scanners, thereby hampering image-guided RT (IGRT). Thus, multiple pre-clinical studies employing LINAC-irradiated volumes involve irradiation of the whole brain, which also includes high doses to normal tissues such as unaffected brain parts, eyes, nose, throat and mouth [12–15].

The lack of suitable radiotherapy applications for pre-clinical studies has provided a market for commercial systems such as the SARPP (Small Animal Radiation Research Platform) and Precision X-Ray, both allowing for highly reproducible and precise IGRT with the smallest circular radiation field size between 0.5–1 mm in diameter [4, 8, 16].

Due to considerable acquisition and running costs, however, many research groups working in the field of radiobiology are not capable to purchase such a system. Our goal thus was to develop an affordable alternative by minimally modifying a commercially available industry micro-CT towards a device capable of IGRT in the setting of murine brain tumors. The costs for these machines are roughly less than a third of the costs of a dedicated small-animal irradiation device, which would allow more groups to include IGRT into pre-clinical models at affordable costs while simultaneously providing extended imaging capabilities in terms of micro-CT [17, 18] as well as digital subtraction angiography [19, 20].

## Materials and Methods

### Micro-CT

We used an industrial micro-CT system (Y.FOX, YXLON GmbH, Hamburg, Germany), which was originally developed for non-destructive material investigations, S1a–S1c Fig. The cabinet includes a flat panel detector (PaxScan 2520D/CL, Varian Medical Systems Inc., Salt Lake City, UT, USA), a CNC manipulator, which can rotate and move objects in micrometer scale and a multifocus transmission X ray tube (FXE.160.51) with acceleration voltages up to 160 kV, emission currents up to 1 A and three different focal spot sizes (1, 3 and 5 μm). The X-ray tube was equipped with a diamond-coated high-power tungsten target and is operating continuously. For commissioning and treatment, we used a beam voltage of 150 kV at a power of 10 W, which resulted in an emission current of 66.7 mA. Due to lack of an exit window, collimation of the beam is feasible directly behind the target. For imaging and irradiation, mice were placed on a couch compatible with the CNC manipulator and immobilized by ear bar fixation.

### Custom Collimators

To collimate the original cone beam to pencil beams suitable for treatment, we used a custom-made combination of a base plate, which can be fixed at the X-ray tube, and 5 cylindrical steel collimators which can be placed in its center (S1d Fig). The drilling has been performed according to the standard ISO 2768-mk, which defines a maximal deviation of ± 0.1 mm for each bore diameter. The manufacturer confirmed the uniformity of the bore diameters in this range. The base plate was made of stainless steel (1.4305/X8CrNiS18-9) and the collimators were made from alloy (1.4301/X8CrNiS18.10) to avoid abrasions of the threads. Acquisition of portal images or CT scans prior to IGRT is feasible with the mounted base plate without insertion of the collimators.

### In-phantom dosimetry

The output of the X-ray tube was measured according to the AAPM TG-61 protocol for 40–300 kV X-ray beam dosimetry in radiotherapy and radiobiology using the in-phantom method [21]. For data acquisition, a cylindrical ionization chamber PTW 31010 (PTW Freiburg GmbH, Freiburg, Germany) and a UNIDOS® E Universal Dosimeter (PTW Freiburg GmbH, Freiburg, Germany) were used. The chamber was placed at the isocenter in the corresponding water-equivalent RW3 slab phantom 29672/U6 (PTW Freiburg GmbH, Freiburg, Germany). For in-phantom dosimetry, two field diameters (5 mm and 4 mm) and an open field were investigated. Due to the partial volume effect of the chamber [4], smaller field sizes were not measured.

### Absolute film dosimetry

Gafchromic EBT3 films (ISP, Wayne, NJ, USA) were used for dosimetry of the various collimators according to the guidelines of the AAPM Task Group 55 [22]. The calibration curve of the films was taken at doses of 0, 0.025, 0.05, 0.1 Gy and in 0.25 Gy steps between 0.25 Gy and 3.0 Gy at the isocenter of the system. The film size was 3x3 cm² and the films were placed at a depth of 5 mm between water-equivalent RW3 slabs. The films were scanned as a 48-bit color image with a resolution of 600 dpi and 16-bit per color channel using an Epson Expression 10000 XL (SEIKO Epson CORPORATION, Suwa, NGN, Japan). Due to the low doses we used the calibrated information of the red color channel [23, 24] (S1 Text). The position of the film at the center of the scanner was marked together with the scan orientation of the films [23]. Scanning was performed after 12 h from irradiation to prevent uncertainties due to the self-developing effect of the films [23].

### Assessment of dose rates in relation to field diameter and depth

We chose EBT3 films for dosimetry as ionization chambers exhibit a partial volume effect at small field sizes [22, 24]. The dose rates for the open field and for collimator aperture sizes of 4 and 5 mm were measured in a water-equivalent RW3 slab phantom corresponding to a depth of 3.2 mm. The dose rates in relation to depth were measured for collimator aperture sizes of 1 mm to 5 mm with EBT3 films with a size of 3x3 cm² at depths of 1, 5, 10 and 20 mm. The films were placed between water-equivalent RW3 slabs and each film was irradiated separately for every depth level. The top of the phantom was always placed at the isocenter.

### Relative Dosimetry

Full width at half maximum (FWHM), flatness, symmetry and penumbra were measured with EBT3 films at depths of 1, 5, 10, and 20 mm. The top of the water-equivalent RW3 slab phantom, which had a total thickness of 30 mm, was set to the isocenter. The analyses were performed following the guideline of the AAPM protocol TG-45 [25] (S2 Text).

### Spatial accuracy of the manipulator

The accuracy of the CNC manipulator carrying the couch was validated in two tests. The first validation considered the positioning in the x/y-plane of the system. A film was placed on top of RW3 slabs at the isocenter and nine independent beams using the collimator with the 1 mm aperture size and a fixed distance of 12 mm from field center to field center should produce a square pattern. The deviation of the beam centers from the expected 12 mm distance was considered as a measure of spatial accuracy. The second validation performed was an off-axis beam test using the collimator with a 3 mm aperture size with five beams. The film was placed between a head phantom of RW3 slabs perpendicular to the beam and was rotated in 72° steps to achieve a star pattern. The angles between every beam and the distances of the corners of the 2 largest stars (resembling 2 pentagons) were measured. At the measurement of the star pattern the standard deviation of the length of the edges was considered as a criterion for symmetry of the rotation.

### Orthotopic mouse model

All animal experiments were conducted according to the German law for the care and welfare of animals and were approved by local authorities (Regierungspräsidium Karlsruhe, 35-9185.81/G-184/14). The human glioblastoma cell line U87MG (ATCC, LGC Promochem, Wesel, Germany) was grown in DMEM medium with 10% FBS (FBS; Biochrom AG, Berlin Germany) under 5% CO2 at 37°C. Immunodeficient 6–8 weeks old female mice (NOD.Cg-*Prkdc* ^*scid*^ *Il*2*rg* ^*tm*1*Wjl*^/SzJ, The Jackson Laboratory, USA) were used for orthotopic tumor implantation. Prior to surgery, the mice were anesthetized by subcutaneous injection of MMF (0.5 mg/kg medetomidine, 5 mg/kg midazolam and 0.05 mg/kg fentanyl) and positioned in a stereotactic frame (TSE Systems, Bad Homburg, Germany) under an operating microscope. After drilling of a 0.5 mm burr hole 1 mm anterior to the bregma and 3 mm lateral to the midline, 2x10^6^ U87MG cells were injected with a glass syringe (Neuros Syringe, Hamilton) using a 33G blunt needle over approximately 10 minutes. For postoperative pain management all animals received 200 mg/kg metamizole. All mice were sacrificed if neurological symptoms were noted.

### CT scanning

All xenografted animals were screened for intracranial tumors once per week. To this end, 300 μl of a iodine-based contrast agent (Iomeprol; Imeron 300, Bracco Imaging Group, Germany) were injected via the lateral tail vein and CT-imaging was performed under s.c. anesthesia using the following scan parameters: tube voltage 80 kV; (current 75 μA), 360° rotation within 33 s scan time and continuous image acquisition at 30 frames per second (resulting in 1000 projections). The imaging procedure applies a total dose of 0.5 Gy per scan to the animals. For reconstruction of RAW-data a filtered back projection algorithm with a matrix of 512 x 512 x 512 was used (Reconstruction Studio, TeraRecon, Foster City, CA, USA) and produces CT images with a pixel size of 40x40x53 *μm*.

### Radiation therapy planning

For treatment planning, we developed an in-house C++ application including a graphical user interface designed in Qt 5.3.1 (Qt Project Hosting, Oslo, Norway). For every beam angle the user has to provide the depth of the reference point in the planning target volume out of the CT images. The underlying application calculates the superposition of the individual beams at the reference point based on the central axis depth dose curves and the field geometries. The user receives the treatment time for every beam as well as the couch position, which has to be approached manually. The evaluation of the planning system was performed with an in-house made head phantom of RW3 slabs with a diameter of 2 cm. Gafchromic films were placed between the head phantom slabs and irradiated with the planned beams of the treatment system.

### IGRT

Prior to irradiation, the base plate was mounted on the X-ray tube and a CT scan with i.v. contrast agent was performed under anesthesia. Next, the depth of each tumor was measured for each planned irradiation angle on the reconstructed CTs. All data was entered into the planning software and the couch was placed according to the positions and angles given by the application. Following mounting of the suitable collimator (depending on the tumor diameter), irradiation was initiated for a period of time as given by the program. Thereafter, irradiated and unirradiated animals were screened weekly for intracranial tumors using contrast-enhanced (Iomeprol; Imeron® 300, Bracco Imaging Group, Germany) micro-CT scans.

## Results

### Dose rates

**Fig 1:**
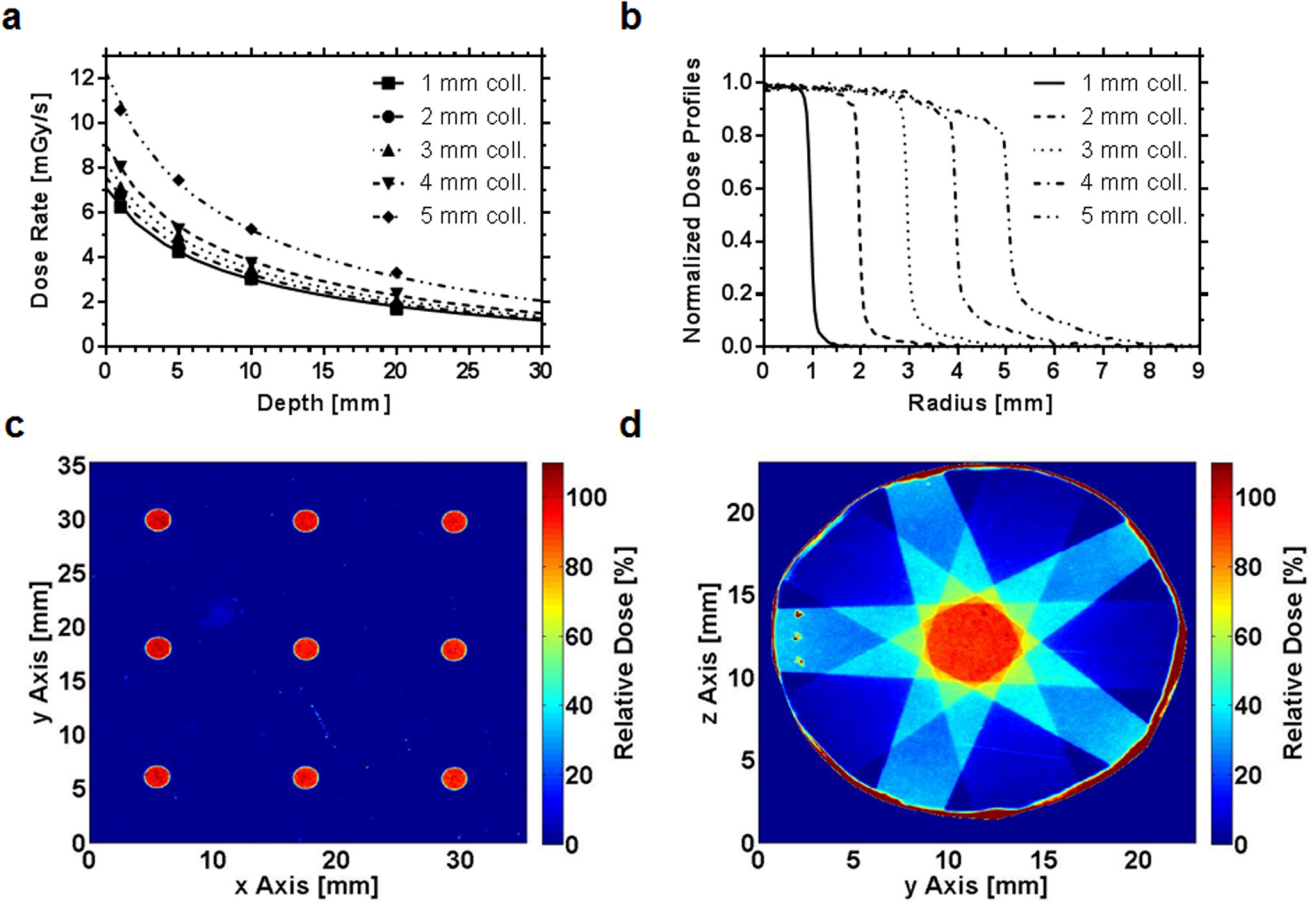
Dosimetric characteristics and reproducibility of the system. (a) Measured data and fitted curves (S3 Text, Eq. (1)) of depth-dose rates. Mean values of three measurements in various depths using all established collimators (aperture sizes 1–5 mm) are shown. (b) Radial dose profiles of the five collimators in 1 mm depth. (c) Accuracy assessment of the manipulator in the x-y-plane. A quadratic pattern was produced and the distances between the spots were measured. (d) Assessment of center of rotation accuracy. A pentagon pattern (right side) was used to assess the angle accuracy of the CNC arm.

The dose rate as a function of the tube power for open fields and collimator aperture sizes of 4 and 5 mm at the isocenter and a depth of 3.2 mm in solid water shows a linear dependence (data not shown). The dose rate increases with tube power and is 7.6 mGy/s and 5.6 mGy/s for collimator aperture sizes of 5 mm and 4 mm in 5 mm depth, respectively. By reducing the collimator aperture size from 5 mm to 4 mm the dose rate decreases by 26%. The depth dose rates for the collimators with 1–5 mm aperture size shows a typical shape of keV photon beams Fig 1a. In 1 and 5 mm depth, the dose rates for the 5 mm collimator were 10.7 mGy/s and 7.6 mGy/s, respectively. By reducing the collimator aperture size from 5 mm to 4 mm the dose rate was decreasing by 24%, 33% for 4 mm to 3 mm, 36% for 3 mm to 2 mm and 41% for 2 mm to 1 mm collimator aperture size.

### Relative Dosimetry

The overall symmetry was 1.04±0.02% (Table 1). A slight heel effect was detected, however, the beam profile shows a homogenous dose in the central region. At the borders of the central region, the beam profile shows a shoulder, leading to a flatness of 4.99±0.63%. The widths of the penumbras ranged from 0.15 to 0.51 mm for the 1 and 5 mm collimator aperture sizes, respectively (Fig 1b). Due to the divergence of the beam the FWHM is increasing with depth, whereas values for symmetry, flatness and penumbra remain constant.

**Table 1:** FWHM, flatness, symmetry, and penumbra for all collimator aperture sizes at a depth of 1 mm measured using EBT3 films.

	**collimator aperture size**				
	1 mm	2 mm	3 mm	4 mm	5 mm
FWHM (mm)	1.97 ± 0.02	4.03 ± 0.00	6.03 ± 0.00	8.03 ± 0.02	9.99 ± 0.02
Flatness (%)	3.29 ± 0.79	4.13 ± 0.91	4.49 ± 0.37	5.28 ± 0.62	7.79 ± 0.48
Symmetry (%)	1.02 ± 0.02	1.04 ± 0.02	1.05 ± 0.02	1.05 ± 0.01	1.05 ± 0.01
Penumbra (mm)	0.15 ± 0.02	0.14 ± 0.02	0.19 ± 0.02	0.36 ± 0.04	0.51 ± 0.04

### Reproducibility of the positioning

Two approaches to assess reproducibility were undertaken (Fig 1c). First, to validate x- and y-axis precision, we measured the distance between nine field centers in x and y direction. Here, the accuracy was in sub-millimeter range for both the x- (±0.11 mm) and y-axis (±0.07 mm). Second, to validate reproducible angle positioning, five beams were used to produce a pentagon with equal edge lengths. Here, the beam angle variation was only minimal (±1.08°) and the variation of the lengths of the edges of the pentagon were in the submillimeter range (±0.27 mm).

### Treatment of intracerebral tumors

**Fig 2:**
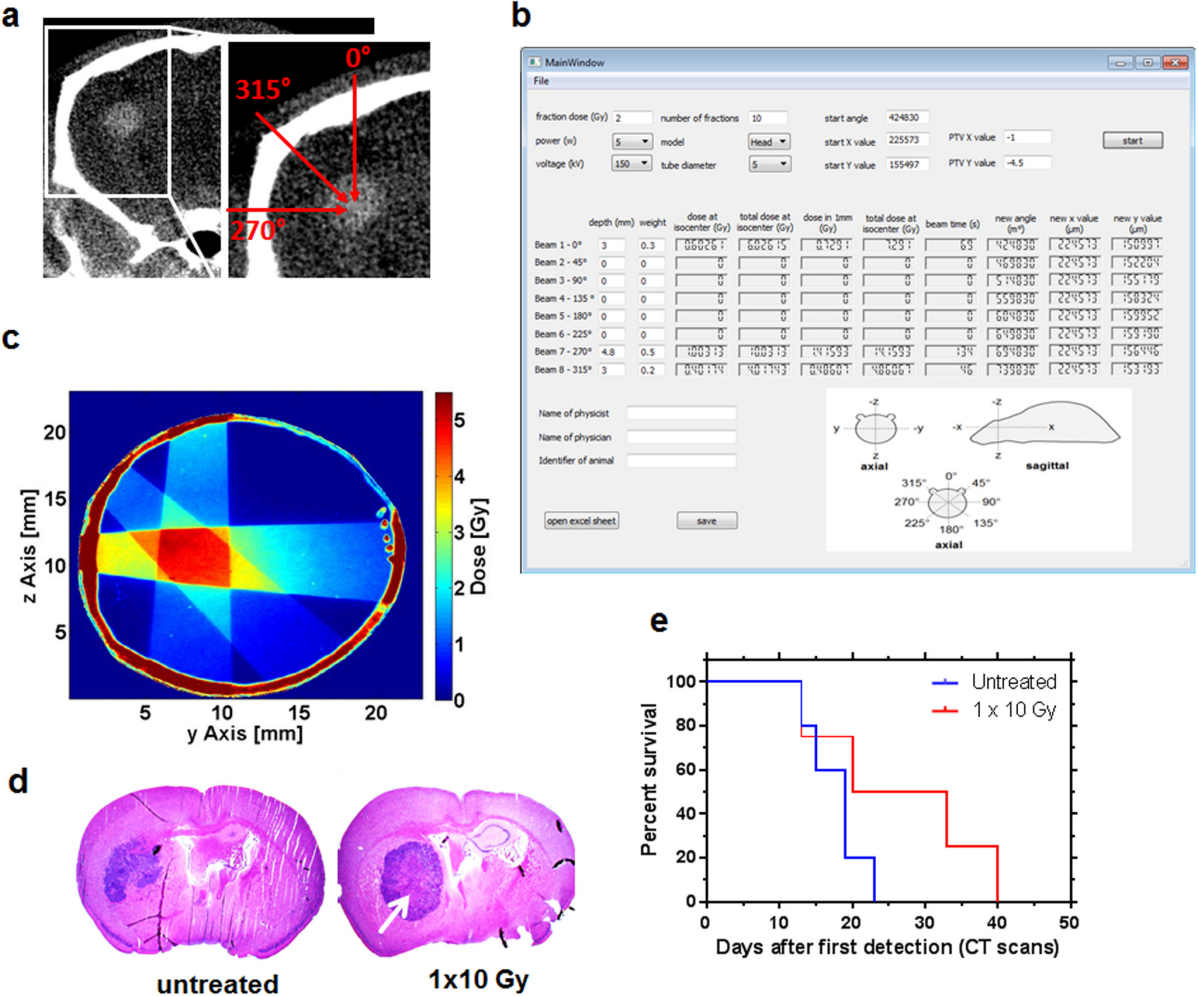
Proof of principle using an orthotopic implant model. (a) CT scans used to measure tumor depths and example for a plan using 3 irradiation angles (0°, 270° and 315°). (b) Graphical user interface of the planning application with a standard treatment scheme. (c) Dose distribution of a three-beam plan (details shown in b) in the head phantom measured with a Gafchromic film. (d) Treated tumors show extensive necrotic areas (white arrow) after irradiation, which were not present in untreated tumors. (e) Survival plots of irradiated and untreated mice.

Prior to irradiation, CT scanning was used to validate intracerebral tumor growth and to assess the various depths for each planned angle measured from the body surface to the tumor center (Fig 2a). The work flow of anaesthetisation, positioning, performing the CT scan, reconstruction of the CT scan, measuring the depth to the tumor and treatment planning took about 20 min. Following treatment planning using the established application (Fig 2b) and validation using radiochromic films (Fig 2c), three mice were irradiated with 10 Gy using the settings calculated in the planning program to ensure adequate target volume coverage. The total treatment time was 19.1 min. Beside mild weight loss along with progressive tumor growth and hair loss in the treatment fields, we did not see relevant adverse events. In turn, post-mortem histological workups of intracranial tumors showed large areas of necrosis, indicating consistent with an efficient antitumoral treatment (Fig 2d). Consistent with this, irradiated mice showed prolonged survival in comparison to untreated mice (Fig 2e).

## Discussion

We here demonstrate that only minimal and inexpensive modifications (roughly in the range of 500 $) are required to convert an industrial CT, which was originally designed for non-destructive material analysis, into an effective small-animal IGRT. In the light of both an increased necessity and a high demand for the implementation of radiotherapy into small animal models, the approach presented here displays an affordable alternative.

One may argue that irradiation may successfully be implemented in small animal tumor models by (heterotopic) implantation in favorable locations that may be easy to irradiate with LINACs or orthovoltage machines (e.g. by subcutaneous implantation in the hind limbs or flanks). While for some tumors, this may result in comparable outcomes, specifically the (radiation) biology of glioblastoma strongly depends on the host tissue microenvironment, which leads to conflicting data with ortho- and heterotopic engraftment models [26, 27]. Another important aspect of pre-clinical irradiation in the setting of glioblastoma is to provide reasonable dose rates to avoid DNA damage repair due to protracted irradiation [28–30]. Our setup provides dose rates of 5.2–8.9 mGy/s in relevant (3–4 mm) depths, resulting in total treatment times of 5.6–10.6 min for 3 Gy and 9.4–17.7 min for 5 Gy. Although these rather narrow time frames are unlikely to result in a bias by increased (sublethal) DNA damage repair, shortening of collimators (e.g. by using high-Z material) would increase the output of the system.

Several alternatives to commercially available machines have been published over the past (reviewed in [9]). Almost all approaches include shaping of the beams either by in-house collimators or by using the pre-formed beams of the manufacturer. Thus, as a logical necessity of highly conformal irradiation is accurate positioning of the animals, the most logical approach is a dual use (imaging and irradiation) of the same X-ray tube. We used a setup suggested by a group from UT Southwestern, consisting of an adaptor ring which is not affecting the cone beam but allowing to reproducibly place the collimators in the central beam [31, 32]. The group equipped a commercially available X-ray cabinet (XRAD 320, Precision X-Ray, Inc.) with a custom collimation system to produce 1–10 mm diameter beams from one angle.

A limitation of our collimation method is the increase of the penumbra proportional to the bore diameter. More sophisticated collimation techniques, such as the method suggested by Graves et al. [1, 2, 5] may help to achieve even higher degrees of conformality (Table 2). However, our system has the advantage of a higher dose rate due to continuous operation mode, which allows faster application if the prescribed dose.

**Table 2:** Comparison between the systems of Mannheim and Stanford [1] for a field size of 1 cm.

	Mannheim	Stanford
system	industrial micro-CT scanner	commercial small animal micro-CT scanner
tube voltage (treatment)	150 kV	120 kV
operation mode	continuous	pulsed
dose rate (mGy/s)	10.7	2.9
FWHM (mm)	9.99	9.2
Flatness (%)	7.79	3.6
Symmetry (%)	1.05	1.0
Penumbra (mm)	0.51	0.45

However, in most small animal models, tumor diameters are commonly relatively small (2–5 mm in our experiments), which will allow using small collimators with negligible penumbras. In summary, we here demonstrate that an affordable industrial micro-CT can be easily and reversibly modified to allow effective small animal IGRT of even delicately located tumors, such as orthotopically implanted glioblastoma in the murine brain.

## Supporting Information

S1 Text Calibration of the Gafchromic EBT3 films. (PDF)

S2 Text Full width at half maximum (FWHM), flatness, symmetry and penumbra. (PDF)

S3 Text Depth Dose Rate Interpolation. (PDF)

S1 Fig Micro-CT and equipment used in this study. (a) Industrial X-ray system YXLON Y.Fox (YXLON International GmbH, Hamburg, Germany). (b) The X-ray tube (A) is mounted at a fixed position on the top of the system. The beam directs towards the manipulator (B), which can be moved in three axes (x,y,z) and rotated by 360°. The beam is detected by a 12-bit direct digital flat panel detector (C). (c) This image shows the X-ray tube outlet with a ring (D) holding the target (E) in place when the system is evacuated. The lower picture shows the animal couch with anesthesia support (F) and ear bar fixations (G). (d) Mounted base plate (left side) and collimator (right side).
(TIF)

S2 Fig Reproducibility of positioning. (a) Uniformity test: irradiated Gafchromic film using all collimators successively. (b) Geometrical reproducibility of a treatment plan with two beams in two different films. (c) Comparison of the length of the dose edges from (b). (d) Reproducibility of the fractional dose with three beams. Left: 118.3 cGy, Right: 115.0 cGy. (e) Reproducibility of the fractional dose with four beams. Left: 118.4 cGy, Right: 119.8 cGy. (f) Reproducibility of the fractional dose with five beams. Left: 115.7 cGy, Right: 115.0 cGy.
(TIF)

S1 Data Data of Fig 1a. (XLSX)

S2 Data Data of Fig 1b. (XLSX)
