# Supplementary material for: Image-Guided Radiotherapy Using a Modified Industrial Micro-CT for Preclinical Applications
Source: PLoS One. 2015 May 19;10(5):e0126246. doi: 10.1371/journal.pone.0126246 (PMC4438006; doi:10.1371/journal.pone.0126246)
Supplement: S1 Text — (PDF) [file pone.0126246.s001.pdf]

S1 Text

Calibration of the Gafchromic EBT3 films. Gafchromic EBT3 films (ISP, Wayne, NJ, USA) were calibrated using the information of the red color channel [1,2]. Scanning was performed after 12 h post irradiation to prevent uncertainties due to the self-developing effect of the films [1,2]. The dependence of the red color channel levels for the calibration curve (CC) on dose D were fitted to the data using a nonlinear least squares algorithm and Eq. (1) with fitting parameters a, b, and c:

$$CC = a \cdot log(b + D) + c \tag{1}$$

References

1. Micke A, Lewis DF, Yu X. Multichannel film dosimetry with nonuniformity correction. Med Phys 2011;38:2523-2534.

2. Schneider F, Polednik M, Wolff D, Steil V, Delana A, Wenz F, Menegotti L. Optimization of the Gafchromic™ EBT protocol for IMRT QA. Z Med Phys 2009;19:29-37.
