# Supplementary material for: Image-Guided Radiotherapy Using a Modified Industrial Micro-CT for Preclinical Applications
Source: PLoS One. 2015 May 19;10(5):e0126246. doi: 10.1371/journal.pone.0126246 (PMC4438006; doi:10.1371/journal.pone.0126246)
Supplement: S2 Text — (PDF) [file pone.0126246.s002.pdf]

S2 Text

Full width at half maximum (FWHM), flatness, symmetry and penumbra. The analysis was performed following the guideline of the AAPM protocol TG-45 [1]. All beam profiles were normalized to the dose maximum and the FWHM was defined as the distance between the 50 % dose points. The flatness is defined along 80 % of the cross-section area and gives its maximum dose variation, according to

$$Flatness = \frac{Dmax - Dmin}{Dmax + Dmin} \tag{1}$$

The deviation of the "left-side" dose and the "right-side" dose at 80 % of the FWHM points describes the symmetry of a field

$$Symmetry = max(|D(x) - D(-x)|) \tag{2}$$

The width of the penumbra is defined between 80 % and 20 % of the maximum dose as the lateral distance on both sides of the beam profile.

FWHM, flatness, symmetry and penumbra were determined for angles of 0°, 45°, 90° and 135°. Mean and standard deviations were calculated.

References

1. Nath R, Biggs PJ, Bova FJ, Ling CC, Purdy JA, van de Geijn J, Weinhous MS. AAPM code of practice for radiotherapy accelerators: Report of AAPM Radiation Therapy Task Group No. 45. Med Phys 1994;21:1093-1121.
