# Supplementary material for: Image-Guided Radiotherapy Using a Modified Industrial Micro-CT for Preclinical Applications
Source: PLoS One. 2015 May 19;10(5):e0126246. doi: 10.1371/journal.pone.0126246 (PMC4438006; doi:10.1371/journal.pone.0126246)
Supplement: S3 Text — (PDF) [file pone.0126246.s003.pdf]

S3 Text

Depth Dose Rate Interpolation.

The dose rate $\dot{D}_{calc}$ can be interpolated by fitting Eq. (1) to the measured data using a nonlinear least squares algorithm:

$$\dot{D}_{calc} = \frac{A}{(D+d)^2} \cdot exp(-\mu(d) \cdot d) \tag{1}$$

with the entrance dose rate A, the known distance $D = 72mm$ of the source to the surface of the solid water slabs, the depth d in tissue (simulated by solid water slabs) and the effective attenuation coefficient $\mu(d)$

$$\mu(d) = \frac{C \cdot \mu_0 + d \cdot \mu_1}{(C+d)} \tag{2}$$

The change of the effective attenuation coefficient is described by a fit parameter C which governs the transition of the effective attenuation coefficient from a (fitted) initial effective attenuation coefficient μ_0 to the known attenuation coefficient of $\mu_1 =$ 0.015052 / mm for infinite depth d, when only the 150 keV photons contribute due to beam hardening.

Note that during fitting the parameters μ_0 and C were shared for all collimator aperture sizes, i.e. only the output factors A were different between the different collimators. Least squares fit was performed using GraphPad Prism (Version 6.05 for Windows, GraphPad Software, La Jolla California USA).
