# Supplementary figures and images for: Image-Guided Radiotherapy Using a Modified Industrial Micro-CT for Preclinical Applications
Source: PLoS One. 2015 May 19;10(5):e0126246. doi: 10.1371/journal.pone.0126246 (PMC4438006; doi:10.1371/journal.pone.0126246)

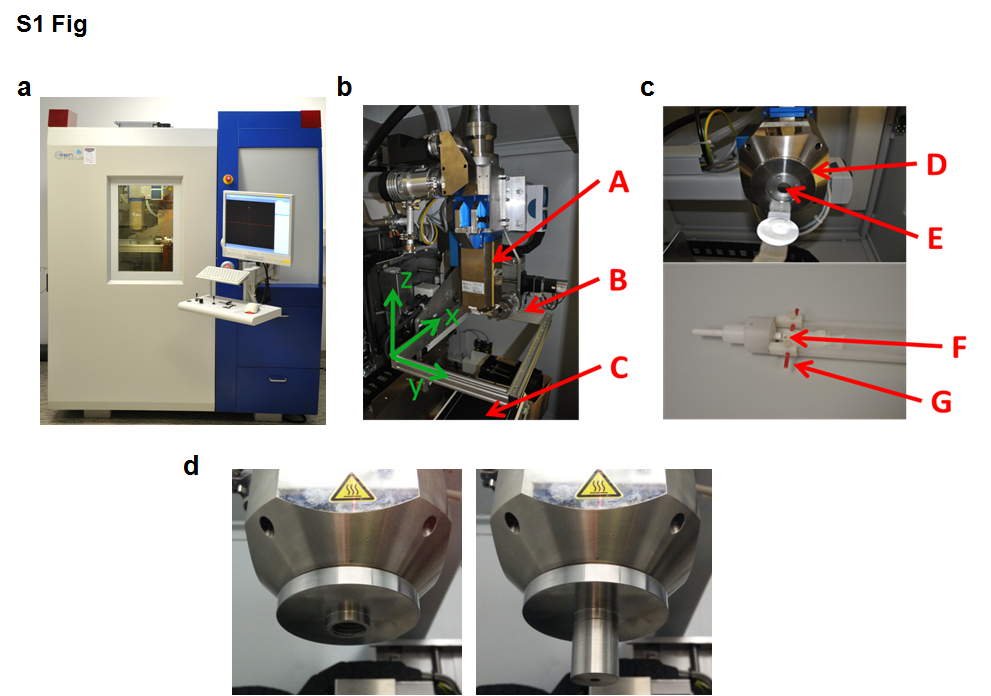

Supplement: S1 Fig — (a) Industrial X-ray system YXLON Y.Fox (YXLON International GmbH, Hamburg, Germany). (b) The X-ray tube (A) is mounted at a fixed position on the top of the system. The beam directs towards the manipulator (B), which can be moved in three axes (x,y,z) and rotated by 360°. The beam is detected by a 12-bit direct digital flat panel detector (C). (c) This image shows the X-ray tube outlet with a ring (D) holding the target (E) in place when the system is evacuated. The lower picture shows the animal couch with anesthesia support (F) and ear bar fixations (G). (d) Mounted base plate (left side) and collimator (right side). (TIF) [file pone.0126246.s004.tif]

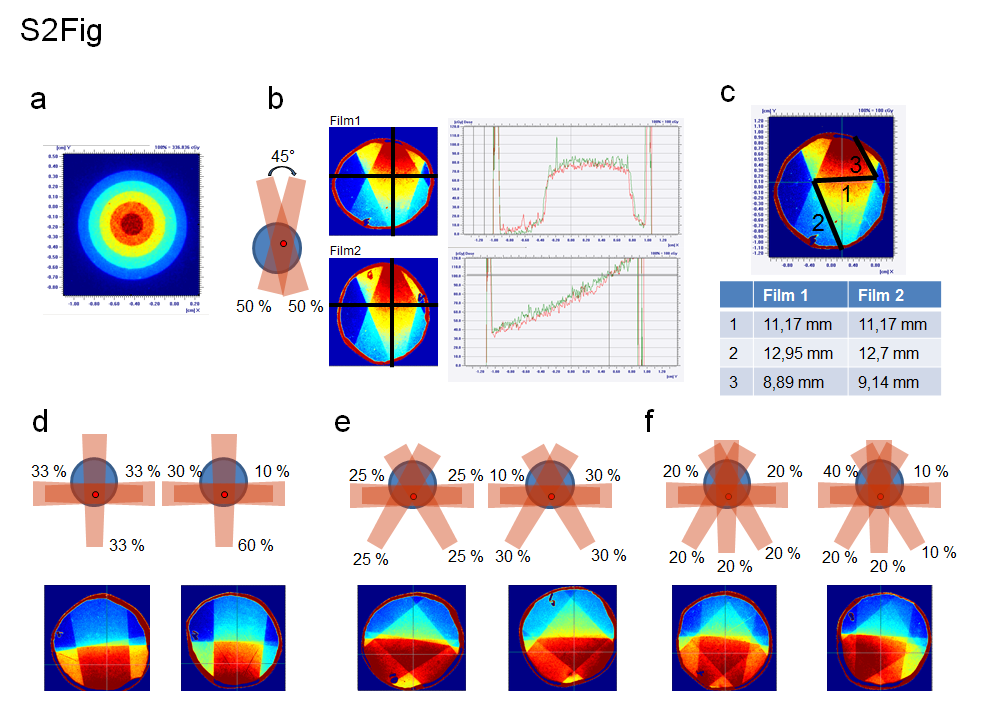

Supplement: S2 Fig — (a) Uniformity test: irradiated Gafchromic film using all collimators successively. (b) Geometrical reproducibility of a treatment plan with two beams in two different films. (c) Comparison of the length of the dose edges from (b). (d) Reproducibility of the fractional dose with three beams. Left: 118.3 cGy, Right: 115.0 cGy. (e) Reproducibility of the fractional dose with four beams. Left: 118.4 cGy, Right: 119.8 cGy. (f) Reproducibility of the fractional dose with five beams. Left: 115.7 cGy, Right: 115.0 cGy. (TIF) [file pone.0126246.s005.tif]
